# Leakage-Aware Retrofit Monitoring for Collaborative Robot Anomaly Detection

**DOI:** 10.3390/s26216746

**Published:** 2026-10-25

**Authors:** Francesco Aggogeri, Kanstantsin Miatliuk, Nicola Pellegrini

**Affiliations:** 1 Department of Mechanical and Industrial Engineering, Università degli Studi di Brescia, Brescia 25123, Italy; nicola.pellegrini@unibs.it; 2 Department of Dynamic Systems, Faculty of Mechanical Engineering, Bialystok University of Technology, 15-351 Białystok, Poland; k.miatliuk@pb.edu.pl

**Keywords:** collaborative robot, inertial measurement unit, anomaly detection, condition monitoring, sensor-channel selection, temporal persistence

## Abstract

External inertial sensing enables controller-independent monitoring of collaborative robots. Robust evaluation should distinguish classifier discrimination from temporal post-processing and explicitly control temporal leakage, sensor–label misalignment, and installation-sensitive sensing shortcuts. This study investigates the UR3e/Nicla Sense ME record using complete-event chronological separation, transition masking, training-only transformations, same-split sensor-channel ablation, raw per-window Random-Forest (RF) scores, and validation-selected H-of-K persistence. Two velocity-anomaly episodes are excluded from all model, threshold, channel, and persistence selection. The same-split ablation selects a gyroscope-only 31-feature RF representation. Before persistence, the held-out RF yields TN = 153, FP = 231, FN = 0, and TP = 384 (MCC = 0.4988), showing high anomaly recall but poor nominal specificity. Validation selects a 5-of-5 persistence rule, yielding TN = 382, FP = 2, FN = 8, and TP = 376, with accuracy = 0.9870, precision = 0.9947, recall = 0.9792, F1-score = 0.9869, and MCC = 0.9741. Both held-out anomaly episodes are detected with an approximately 40 s confirmation delay after the first eligible positive window. The 21-of-21 rule is retained as a consistency reference and reproduces TN = 384, FP = 0, FN = 40, and TP = 344, with approximately 200 s confirmation delay. Guard-band, persistence, sensor-channel, rolling-origin, protocol-component ablation, and same-split comparator analyses show that performance depends on the evaluation protocol and held-out event. The evidence supports bounded later-event recognition within the same robot and acquisition domain; future studies should establish the long-term false-alarm rate and test cross-platform generalization.

## 1. Introduction

Collaborative robots are increasingly deployed in flexible manufacturing, inspection, laboratory automation, assembly, and machine tending. Their operating signatures depend on mechanical condition and on trajectory phase, programmed speed, payload, tool interaction, sensor mounting, and the local electromagnetic environment. Retrofit condition monitoring therefore requires robust sensor interpretation rather than a single fixed threshold. External inertial measurement units (IMUs) can be installed without privileged access to proprietary servo channels and can capture acceleration, angular rate, vibration, and task periodicity. Their diagnostic value, however, depends on the acquisition stream, label source, and validation design.

The data precedent is CASPER [1,2], a context-aware anomaly-detection system for industrial robotic arms based on external inertial measurements and robot-side annotations. These studies establish the public nine-axis record used here and make the separate acquisition paths central to interpretation. Other work addresses operating context and adaptation: TWINE [3] links digital-twin and explainable-AI functions; Polenghi et al. [4] combine series modeling with hybrid AI for fault diagnosis in articulated collaborative robots; Kermenov et al. [5] address concept-drift adaptation in a dynamic collaborative-robot setting; and Ayankoso et al. [6] examine condition monitoring under trajectory changes. Reviews of fault diagnosis for industrial robots and multi-axis machines and of deep-learning methods for rotating machinery [7,8] map the wider diagnostic field. Together, these studies establish context-sensitive monitoring as a recurring need. Their distinct system aims and evidence bases do not provide direct score comparisons with the velocity-deviation evaluation reported here. Predictive-maintenance mapping across Industry 4.0 sectors [9] situates robot monitoring in a broader maintenance context. Robot-specific acoustic and vibration studies demonstrate reconstruction-based pipelines: Yun et al. [10] learn normal sound from a robot arm with an autoencoder, while Zhong et al. [11] use a sliding-window convolutional autoencoder on vibration signals. Park et al. [12] target programmable motion faults in collaborative robots; Kumar et al. [13] apply transfer learning to servomotor-bearing diagnosis; and Yang et al. [14] address missing data and collaborative feature analysis in multivariate robot-joint records. Further work combines a digital twin with a physics-informed hybrid convolutional autoencoder [15], detects robot-base loosening from vibration [16], and applies attention-based and residual neural networks to robot and joint fault diagnosis [17,18]. Recent models use evolutionary search for robot-joint anomaly detection [19], multiscale empirical-mode decomposition with a CNN–BiGRU for gearbox diagnosis [20], and a hybrid deep-learning framework for industrial-robot faults [21]. These contributions span different sensors, fault mechanisms, and diagnostic targets; they motivate the present study but their headline metrics are not direct benchmarks for this dataset. The methodological comparison clarifies the evaluation gap. CASPER [1,2] creates anomalies through scheduled joint-velocity changes and notes possible label mismatch because the robot-side controller stream and edge-mounted IMU are acquired separately; its detector uses sliding-window scores and thresholding. Polenghi et al. [4] report 1000 healthy cycles for training and 200 for testing, treat a fourth trajectory as an unknown test condition, and use reconstruction-error thresholds for health-state identification. These choices address their respective aims. The present protocol adds complete-event holdout, masks uncertain transition windows, and selects persistence on validation before reporting raw and post-persistence outcomes on the same test events. This comparison identifies complementary controls without implying that earlier studies lack sound safeguards. Broader time-series anomaly-detection reviews [22,23,24] and evaluation studies [25,26,27,28] discuss model families, split design, event-sensitive scoring, and metric choice under class imbalance. This literature motivates reporting raw classifier scores separately from temporally filtered alarms and interpreting results at the event level. The reported protocol tests those evaluation choices within the CASPER UR3e record.

The contribution is an auditable, dataset-specific evaluation protocol for external-IMU collaborative-robot monitoring. It quantifies five sources of performance distortion: temporal window leakage, uncertain sensor–label transitions, future-data contamination during preprocessing, installation-sensitive sensor channels, and temporal alarm filtering. Complete anomaly episodes are held out chronologically; a predefined guard band excludes uncertain transitions; data-dependent transformations and model choices are selected on development data only; channel subsets use the same split; and raw scores are reported separately from persistent alarms. The scientific result is the measured separation between classifier discrimination and decision-layer performance, together with evidence of event-to-event heterogeneity and channel dependence. Claims are bounded to later velocity-anomaly recognition within one robot and acquisition domain; component-fault diagnosis, long-term false-alarm performance, and cross-robot transfer are not established.

### Research Questions

Five research questions structure the revised evaluation. RQ1 asks how complete-event chronological evaluation differs in interpretation from a window-stratified audit. RQ2 asks which IMU channel subset is selected when acceleration, gyroscope, magnetic, and combined representations are compared under the identical chronological split. RQ3 asks how the raw RF evidence is transformed by alternative H-of-K persistence rules in terms of false alarms, recall, MCC, and confirmation delay. RQ4 asks how sensitive the conclusions are to transition guard width and earlier rolling-origin event folds. RQ5 asks whether alternative model families evaluated on the same event split change the methodological conclusion, and which claims remain statistically defensible when only two final anomaly episodes from one robot constitute the independent held-out test.

## 2. Materials and Methods

### 2.1. Dataset and Experimental Context

**Figure 1:**
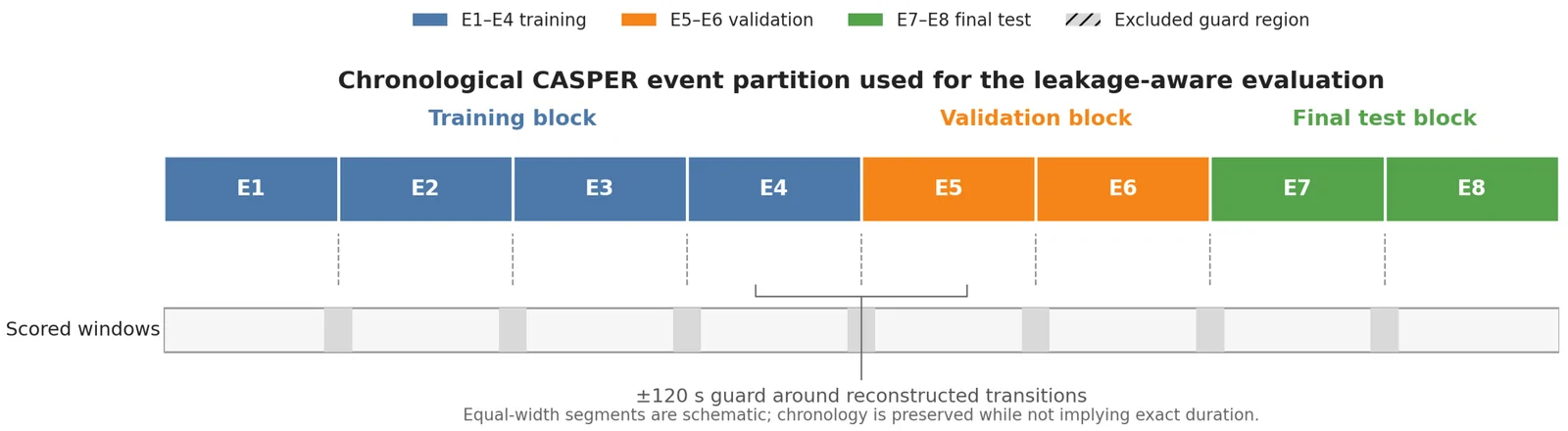
Chronological CASPER partition used for training, validation, and final testing.

This study uses the public Industrial Robotic Arm—IMU Data (CASPER 1 & 2) repository [1,2]. The primary record contains measurements from a Nicla Sense ME mounted on a Universal Robots UR3e platform. The external-sensor record contains 1,751,682 valid observations of AccX, AccY, AccZ, GyroX, GyroY, GyroZ, MagX, MagY, and MagZ. Following the public preprocessing logic, 897 initial idle samples are removed and the analysis is truncated to 1,728,006 aligned samples. The robot-side stream contains the binary ‘Anomaly State’ annotation and kinematic/controller variables; these controller variables are not used as classifier inputs. Figure 1 summarizes the aligned long-record chronology and the immutable E1–E4/E5–E6/E7–E8 development split used throughout the primary analysis. Table 1 summarizes the data resources and their analytical roles.

The public CASPER procedure [1,2] organizes the long record around eight repeated velocity-anomaly episodes, each 36 min in the published schedule. After applying the stated 11,059-sample alignment correction, the nominal elapsed-time intervals are E1 [890.8, 926.8), E2 [962.8, 998.8), E3 [1034.8, 1070.8), E4 [1106.8, 1142.8), E5 [1178.8, 1214.8), E6 [1250.8, 1286.8), E7 [1322.8, 1358.8), and E8 [1394.8, 1430.8) min. At the nominal 20 Hz rate, these correspond, respectively, to end-exclusive sample-index ranges [1,068,960, 1,112,160), [1,155,360, 1,198,560), [1,241,760, 1,284,960), [1,328,160, 1,371,360), [1,414,560, 1,457,760), [1,500,960, 1,544,160), [1,587,360, 1,630,560), and [1,673,760, 1,716,960). These indices are nominal conversions of the corrected elapsed-time labels; because the effective sensor cadence is not exactly constant, the corrected time intervals are the primary boundaries. The split assigns E1–E4 to training, E5–E6 to validation, and E7–E8 to the untouched test. CASPER also provides five challenge recordings representing earthquake-like vibration, direct arm impact, platform impact, added gripper mass, and magnetic-field disturbance, together with alternative 2, 10, and 20 Hz collections. The sampling-rate variants are not used to claim sampling-rate robustness.

### 2.2. Measurement Representation, Alignment, and Label Band

At sample k, the external sensor state is represented by the nine-dimensional vector in Equation (1):

x_k_ = [a_k_^T^, ω_k_^T^, m_k_^T^]^T^ ∈ ℝ^9^ (1)

where a_k_, ω_k_, and m_k_ denote the three-axis acceleration, angular-rate, and magnetic-field vectors, respectively. The dataset reports effective acquisition rates that can deviate from the nominal 20 Hz rate and applies an 11,059-sample correction during label reconstruction [1,2]. The present analysis uses the published correction as the nominal alignment reference. The residual clock and transition uncertainty cannot be treated as exact row-wise ground truth; label transitions are protected by an exclusion region and are not used for onset-latency claims. The long record is divided into windows that do not cross a declared discontinuity or a protected label-transition region. For a window of L samples and stride h, the segmentation rule is defined in Equation (2):

W_i_ = {x(k): s_i_ ≤ k ≤ s_i_ + L − 1},   s_i_ = 1 + (i − 1)h (2)

Windows are generated only after the chronological partition has been declared. The primary analysis uses L = 200 samples and h = 200 samples, so adjacent scored windows do not overlap; at the nominal 20 Hz rate, each represents approximately 10 s. With the primary ±120 s transition guard, any window intersecting a protected transition, a data discontinuity, or a mixed-label interval is omitted before feature extraction and scoring. Omitted windows are not inserted as negative votes. Instead, the persistence state is initialized empty at the start of each contiguous retained segment and at each split boundary. The per-window manifest’s segment identifiers reproduce the stored gyroscope-only 5-of-5 and 21-of-21 decisions exactly when used to reset history; this check matches all 8255 retained score rows. Across the E7/E8 test transitions, the last retained nominal window and first retained anomalous window are separated by 4800 aligned samples (240 s, or 24 nominal window slots); the state is reinitialized when scoring resumes. Because the masked transitions and segment boundaries are derived from the annotated event schedule, this is a retrospective event-aware evaluation and does not demonstrate a continuous online alarm stream that can be generated without anomaly-boundary knowledge. Any deployment would require a reset or missing-data rule based only on signal continuity or data-quality status. To assess sensitivity to masking, the full procedure is also explored with guard half-widths of 0, 30, 60, and 120 s. Even the 0 s sensitivity setting is not treated as a precise physical onset-latency experiment because alignment remains uncertain.

### 2.3. Feature Extraction and Training-Only Scaling

Each accepted 200-sample window is converted into physically interpretable descriptors. For each three-axis sensor family, the feature interface contains axis-wise mean, standard deviation, root mean square (RMS), peak-to-peak range, skewness, Fisher kurtosis, and mean squared first-difference energy; the same seven descriptors are computed for vector magnitude, and three within-sensor axis correlations are added. This produces 31 features per sensor family, 62 for acceleration+gyroscope, and 93 for all nine IMU channels. Five supervised channel sets are evaluated under the identical chronological split: acceleration only, gyroscope only, acceleration+gyroscope, magnetometer only, and acceleration+gyroscope+magnetometer. Standardization is fitted on training windows only and frozen thereafter. Validation-only ablation selects the 31-feature gyroscope-only representation, which is therefore used for the primary RF results. Training-only standardization is defined in Equation (3).

(3) $$\tilde{z}(i,j)=[z(i,j)-\mu (j,\mathrm{tr})]/[\sigma (j,\mathrm{tr})+\varepsilon ]$$

where μj,tr and σj,tr are the training-set mean and standard deviation for feature j. Estimating them from validation or test windows would allow information to influence the model. The complete feature interface is stated in Equation (4):

z(i) = Φ(W(i)) = [f(i,1), f(i,2), …, f(i,p)]^T^ (4)

A useful orientation-robust descriptor is the acceleration magnitude defined in Equation (5):

(5) $$Magnitude\left(a\left(k\right)\right)\;=\;\sqrt{AccX{\left(k\right)}^{2}+AccY{\left(k\right)}^{2}+AccZ{\left(k\right)}^{2}}$$

with an analogous expression for angular-rate magnitude. Magnetic magnitude is calculated for the sensor-dependence audit and distribution-shift analysis. The validation-selected primary supervised model uses gyroscope-derived descriptors only; acceleration and magnetic channels are retained exclusively for the same-split ablation analyses.

### 2.4. Supervised Random-Forest Evidence

The supervised branch estimates whether a feature vector resembles the reviewed abnormal class. Random Forest is used because it can model nonlinear interactions without requiring a high-capacity latent representation and because feature importance remains accessible for engineering inspection. For node S, the Gini impurity is defined in Equation (6): The Random-Forest formulation follows the original ensemble framework [29].

G(S) = 1 − [p(class 1)^2^ + ⋯ + p(class C)^2^] (6)

and the deployed abnormal-class probability is the average over B trees, as defined in Equation (7):

(7) $$p_{\mathrm{RF}}(i)=[p_{1}(y=1|\tilde{z}(i))+\cdots +p_{B}(y=1|\tilde{z}(i))]/B$$

The Random Forest uses 100 trees, maximum depth 8, the Gini criterion, class_weight = ‘balanced’, default square-root feature subsampling, and random_state = 42. A StandardScaler is fitted on training windows only. The forest is trained on chronological events E1–E4. The abnormal-class probability threshold is selected on E5–E6 by exhaustive validation search to maximize MCC, with lower false-positive rate and then higher recall as tie-breakers; the selected threshold for the gyroscope-only model is 0.018884. The complete raw per-window probability vector is retained for the revision analysis. The sensor set, threshold, and persistence parameters are selected using training and validation data only. The preprocessing constants and trained detector are then frozen, and E7–E8 are scored once. Reporting the raw vector separates classifier-level errors from the effects of persistence.

### 2.5. PCA-Based Exploratory Geometry (No Alarm Decision)

PCA is retained only as a label-independent visualization of the exploratory feature geometry. It is fitted to standardized training features, and its loading matrix is frozen before validation and testing. The covariance matrix and eigensystem are given in Equation (8), following the standard principal-component framework [30]. The PC1 and PC2 projection is descriptive and is not used to produce the reported binary detections.

(8) $$C={\tilde{Z}}^{T}\tilde{Z}/(N_{\mathrm{train}}-1),\;\;Cv(q)=\lambda (q)v(q)$$

The PCA analysis is not an operational out-of-distribution detector in this study. No robust-distance threshold, logistic calibration, context-dependent threshold, evidence-fusion coefficient, or PCA alarm-persistence rule is fitted or applied. Accordingly, parameters β and γ from a generic score transformation, as well as α and δ from optional fusion or context formulas, are not empirical settings of this study and are omitted. PCA results are limited to the frozen two-component visualization; no component-level fault diagnosis is inferred from geometric separation.

PCA is not optimized to reproduce the supervised anomaly labels. Its purpose here is to visualize a frozen coordinate system and show whether the exploratory operating-regime geometry aligns with the binary label. Challenge recordings are treated as heterogeneous qualitative stressors; no thresholded OOD-alert count or challenge-file detection rate is claimed. This distinction is essential for impacts, added mass, vibration, and magnetic disturbance, which do not share a common diagnostic taxonomy.

### 2.6. Persistence for the Supervised Detector

The primary binary alarm uses only the supervised gyroscope-feature branch. The RF abnormal-class probability is thresholded using the validation-selected value, and temporal persistence is applied to that binary sequence as defined in Equation (9). PCA is not fused with RF and does not affect the reported alarm.

A_i_ = I[Σ_j=i−k+1_^i^ I(S_j_ > τ_j_) ≥ H] (9)

For the primary binary benchmark, persistence is selected from a validation-only sweep over K ∈ {1,3,5,10,15,21} and H ∈ {1, ceil(K/2), ceil(0.75K), K}, with duplicate H values removed. Post-persistence MCC is the primary selection criterion. The maximum validation MCC is obtained with H = K = 5. An alarm is therefore confirmed after five consecutive positive windows; with non-overlapping 200-sample windows, this adds four update intervals after the first eligible positive window, approximately 40 s at the nominal 20 Hz rate. The previously reported H = K = 21 setting is retained as a sensitivity and consistency reference, not as the primary operating point. It exactly reproduces the earlier final confusion matrix but requires approximately 200 s confirmation. These delays are measured from the first eligible scored positive window, not from physical anomaly onset, because transition uncertainty and the guard policy prevent a defensible onset-to-alarm estimate.

### 2.7. Leakage-Aware Evaluation and Statistical Reporting

The chronological partition is defined by complete anomaly episodes. E1–E4 form training, E5–E6 validation, and E7–E8 the untouched final test. The retained counts are 6676 training windows and 768 validation windows. Although 811 eligible windows are assigned to the final chronological test segment, the defined core E7–E8 test contains 768 scored windows (384 anomalous and 384 nominal); 43 eligible post-E8 tail windows are outside this core test and are not included in its metrics. The 384 nominal controls are exactly window_id 7444–7635 and 7828–8019 (192 windows in each block), representing the two 32 min nominal intervals immediately preceding the guard-cleared E7 and E8 event cores. In the zero-based aligned 1,728,006-sample record, their start-inclusive/end-exclusive sample ranges are [1,546,541, 1,584,941) and [1,632,941, 1,671,341), respectively. All data-dependent operations, including scaling, channel selection, RF fitting, probability-threshold selection, and persistence selection, are completed without E7–E8. Windows remain serially dependent even when non-overlapping; accordingly, the two final anomaly episodes, not the 768 scored windows, are the principal independent units supporting later-event claims. A secondary rolling-origin analysis has been performed on earlier events while leaving E7–E8 fixed: Fold A trains E1–E2, validates E3, and tests E4; Fold B trains E1–E3, validates E4, and tests E5; Fold C trains E1–E4, validates E5, and tests E6. The rolling-origin results are interpreted as evidence of event-to-event heterogeneity rather than as additional independent replications of the final test.

Accuracy, precision, recall, specificity, F1-score, balanced accuracy, and Matthews correlation coefficient (MCC) are reported as descriptive window-level measures. Event-level reporting additionally records the number of anomaly episodes detected, persistent false-alarm events, nominal exposure, and confirmation delay from the first eligible scored positive window. Because only two independent anomaly episodes are present in the final held-out test, no binomial confidence interval based on 768 windows is interpreted as population-level uncertainty. A five-seed stratified 70/15/15 random-window audit uses the same 8255 eligible windows, 31 gyroscope descriptors, training-only StandardScaler, RF settings (100 trees, depth 8, Gini, balanced class weights, square-root feature subsampling, random_state = 42), and validation-MCC threshold-selection criterion as the chronological pipeline. The split seeds are 11, 22, 33, 44, and 55, with 5778/1238/1239 training/validation/test windows per repeat. As in the chronological protocol, each repeat selects its numerical threshold on validation data by maximizing MCC, with lower false-positive rate and then higher recall as tie-breakers. Persistence is not applied to randomly scattered test rows; the partition comparison therefore uses raw classifier results only. Windows from the same complete events can occur in every random subset, so this audit does not test generalization to unseen events.

### 2.8. Robustness, Ablation, and Same-Protocol Analyses

The quantitative analysis contains four robustness components under controlled chronology: a 20-configuration H/K persistence sweep; guard-band sensitivity at 0, 30, 60, and 120 s; same-split sensor-channel ablation; rolling-origin evaluation on earlier events; a one-factor protocol-component ablation; and fully specified alternative model-family comparisons. The final E7–E8 test remains excluded from all development decisions. PCA is descriptive and is not included in the binary detector.

#### 2.8.1. Protocol-Component Ablation Method

The primary reference configuration is chronological E1–E4 training/E5–E6 validation/E7–E8 testing, a ±120 s transition guard, the validation-selected 31-feature gyroscope representation, the validation-selected RF threshold, and 5-of-5 persistence. One factor is then changed at a time while the remaining elements are held fixed whenever the archived outputs permit a paired comparison: persistence is removed; 5-of-5 is replaced by 21-of-21; the transition guard is reduced to 0 s; and the gyroscope representation is replaced by acceleration+gyroscope or all nine IMU axes. A separate five-seed random-window audit is retained as non-paired protocol-sensitivity context because random partitioning changes the statistical estimand and does not support event-level persistence. StandardScaler fitting is restricted to training data for every feature-based model. Because tree split ordering is invariant to positive affine rescaling, scaling is treated as a leakage-control requirement rather than a meaningful RF performance ablation.

#### 2.8.2. Fully Specified Same-Split Comparators

All comparator thresholds are selected on E5–E6 by maximizing raw-score MCC, with lower false-positive rate and then higher recall as tie-breakers; the common 5-of-5 persistence rule is applied only after threshold selection. Random Forest uses the primary 31 standardized gyroscope descriptors, 100 trees, maximum depth 8, Gini impurity, class_weight = ‘balanced’, max_features = ‘sqrt’, min_samples_leaf = 1, and random_state = 42. XGBoost uses the same 31 descriptors with 120 trees, maximum depth 4, learning rate 0.05, min_child_weight = 1, subsample = 0.9, colsample_bytree = 0.9, reg_alpha = 0, reg_lambda = 1, gamma = 0, binary logistic objective, histogram tree method, and random_state = 42. The nominal-trained autoencoder uses the same standardized 31-feature input and architecture 31-16-8-4-8-16-31 with ReLU hidden activations and linear output. It is trained only on nominal training windows using mean-squared reconstruction error, Adam (learning rate 0.001), batch size 64, maximum 40 epochs, and patience 6 on the deterministic final 15% of nominal training windows; the anomaly score is mean feature-wise reconstruction error. The 1D-CNN instead receives raw 3 × 200 gyroscope windows standardized channel-wise from training data only. Its architecture is Conv1D(3→16, k = 7)-BatchNorm-ReLU-MaxPool2, Conv1D(16→32, k = 5)-BatchNorm-ReLU-MaxPool2, Conv1D(32→64, k = 3)-ReLU-global-average-pooling, Dense(64→32)-ReLU-Dropout(0.2)-Dense(32→1). It is trained for 10 fixed epochs with class-weighted binary cross-entropy, Adam (learning rate 0.001, weight decay 1 × 10^−5^), batch size 64, and seed 42. The fixed epoch count avoids validation-based architecture stopping beyond the common threshold selection. The software tools used are scikit-learn 1.8.0, XGBoost 3.1.3, PyTorch 2.10.0+cpu, NumPy 2.3.5, pandas 2.2.3, and SciPy 1.17.0.

## 3. Results

### 3.1. Dataset Audit and Window-Level Descriptors

**Figure 2:**
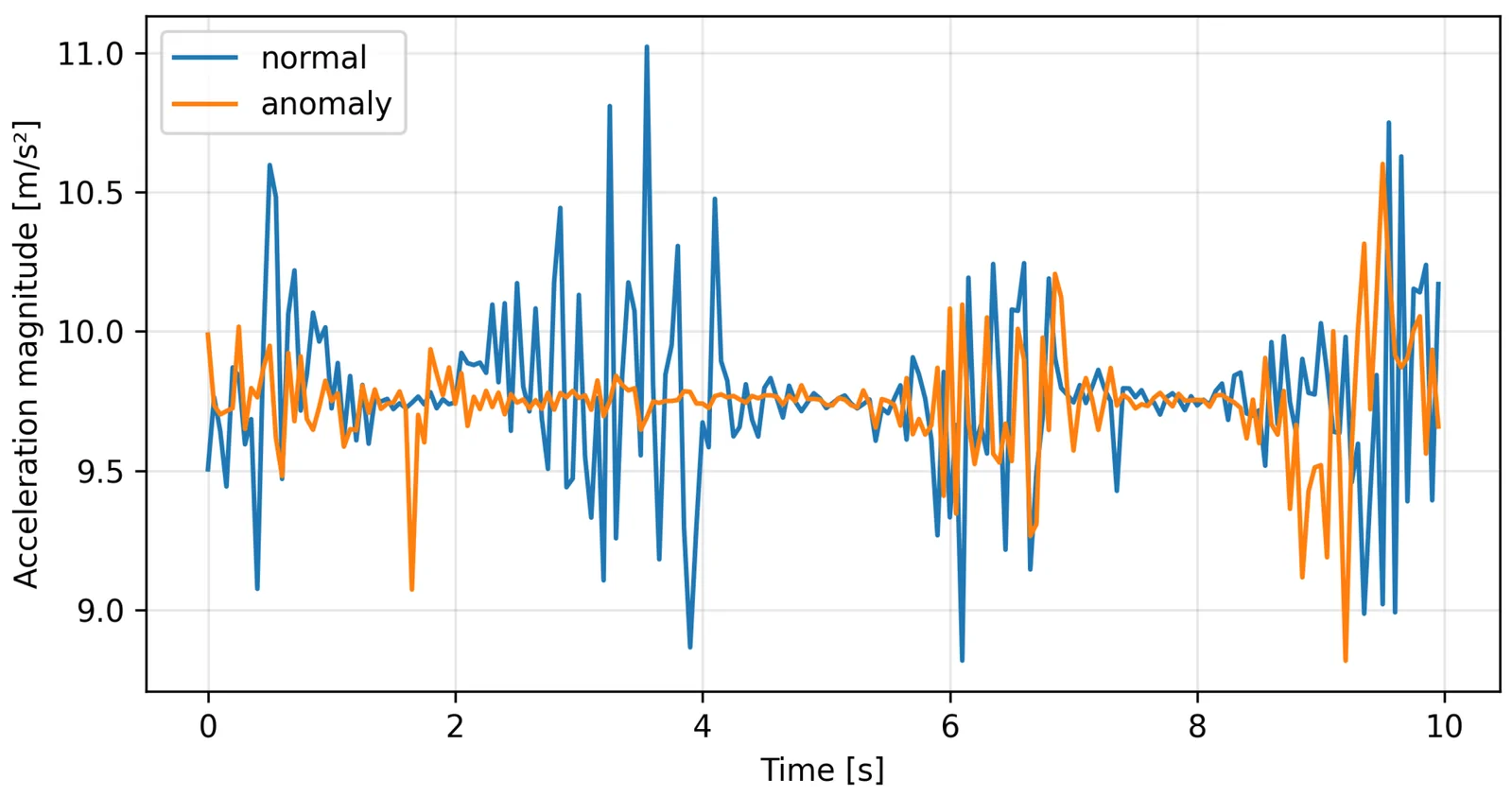
Nominal and velocity-anomaly acceleration excerpts from the exploratory subset.

**Figure 3:**
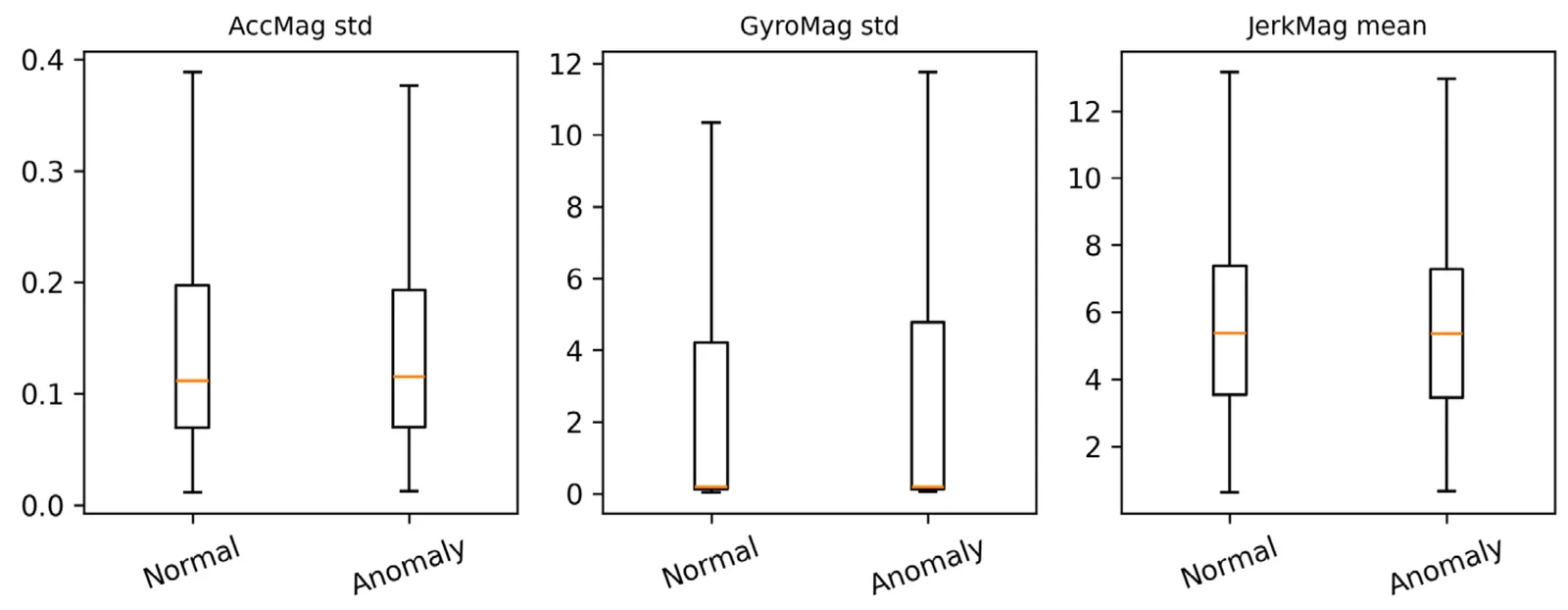
One-second descriptor distributions in the exploratory nominal and anomaly subset.

The binary robot-side annotation contains eight repeated velocity-anomaly episodes, permitting event-aware chronological validation while keeping the final two episodes completely outside model development. Figure 2 shows representative acceleration excerpts, whereas Figure 3 summarizes one-second descriptor distributions from an earlier sequential characterization subset comprising 120,000 samples transformed into 6000 one-second non-overlapping windows (4571 nominal and 1429 labeled anomaly windows). These exploratory plots illustrate measured signal behavior and feature distributions; they are not independent replicates and do not replace the final event-held-out evaluation.

### 3.2. Protocol-Sensitivity Audit

The five-seed random-window audit gives mean test accuracy 0.8988 ± 0.0039, F1-score 0.6762 ± 0.0347, MCC 0.6392 ± 0.0150, recall 0.5723 ± 0.0718, specificity 0.9736 ± 0.0153, ROC-AUC 0.9258 ± 0.0095, and PR-AUC 0.8084 ± 0.0137 (mean ± sample SD). The validation-selected thresholds range from 0.5673 to 0.6318 across seeds. On the identical gyroscope-only RF pipeline, the raw event-held-out E7–E8 test has an accuracy of 0.6992 and an MCC of 0.4988. The random test sets each contain 1239 windows, including 231 anomalous windows (18.6%), whereas the core chronological test is balanced at 384/384. Moreover, random partitions place windows from E7/E8 in training or validation as well as test, so the higher random-split MCC cannot be interpreted as stronger later-event generalization. This matched comparison isolates the partitioning question at classifier level.

### 3.3. Classifier-Level and Persistence-Level Detection Performance

**Figure 4:**
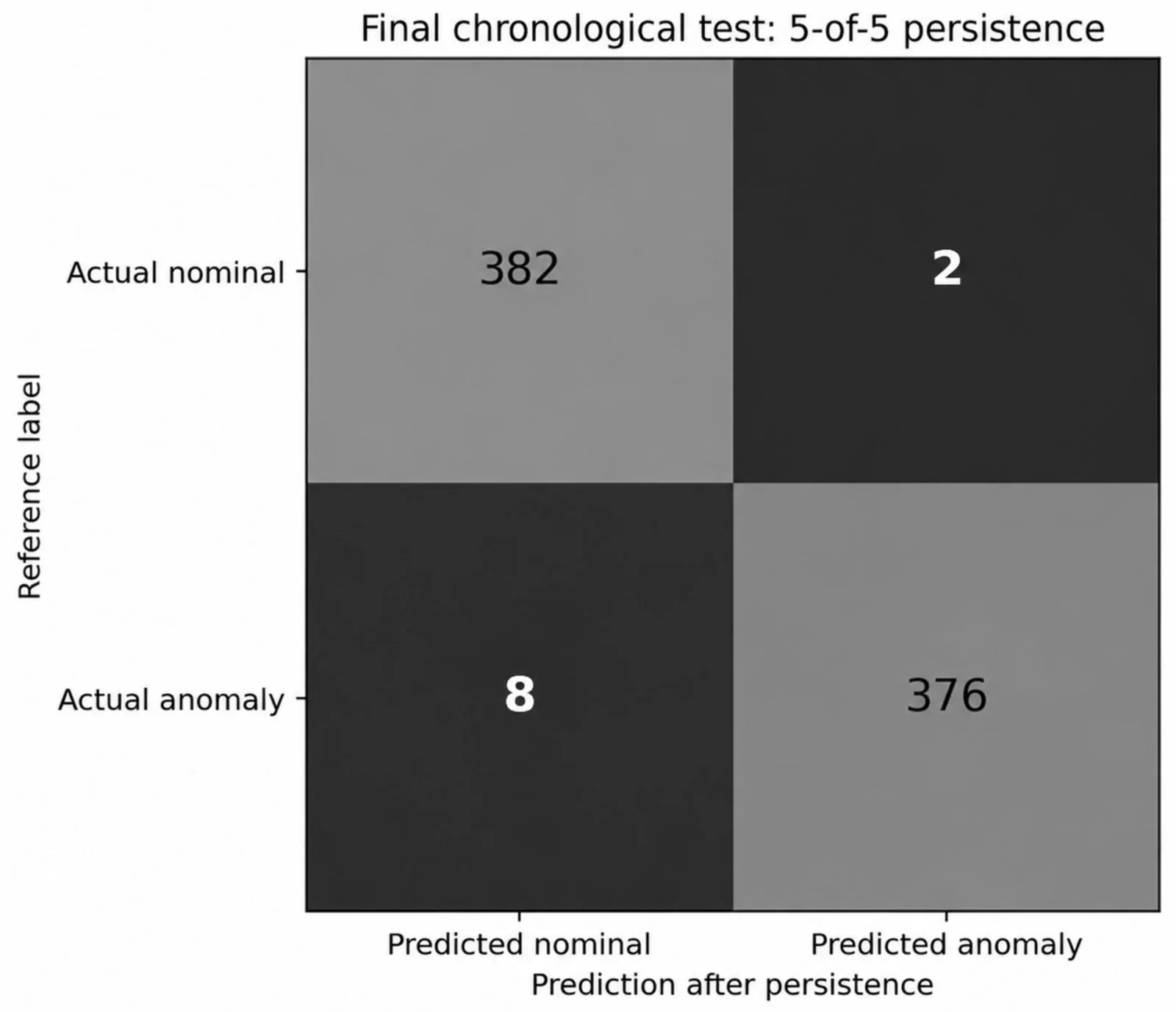
Final-test confusion matrix for the validation-selected 5-of-5 persistence rule.

The final core test contains 768 scored 200-sample windows after the ±120 s guard is applied: 384 nominal and 384 anomalous. At the validation-selected threshold of 0.018884, the raw gyroscope-only RF classifies all 384 anomalous windows as positive but also labels 231 nominal windows positive, giving TN = 153, FP = 231, FN = 0, TP = 384, accuracy = 0.6992, recall = 1.0000, specificity = 0.3984, F1 = 0.7688, and MCC = 0.4988. Thus, the classifier alone is highly sensitive but insufficiently specific. Applying the validation-selected 5-of-5 persistence rule yields TN = 382, FP = 2, FN = 8, TP = 376, accuracy = 0.9870, precision = 0.9947, recall = 0.9792, specificity = 0.9948, F1 = 0.9869, and MCC = 0.9741. Both held-out anomaly episodes are detected, with a median 40 s confirmation delay from the first eligible positive window. The two remaining FP windows form one persistent false-alarm event during 1.0667 h of nominal exposure, an observed rate of 0.9375 events/h that is too imprecise to extrapolate to long-term deployment. Figure 4 visualizes the final post-persistence confusion matrix, while Table 2 records the fixed chronological protocol.

The 11-of-11 setting has been evaluated with the unchanged validation-selected RF threshold (0.018884) and the same segment-reset policy, gives TN = 384, FP = 0, FN = 20, and TP = 364 on the core test (accuracy = 0.9740, precision = 1.0000, recall = 0.9479, F1 = 0.9733, MCC = 0.9492). Each event has ten initial false-negative windows, corresponding to 100 s confirmation from the first eligible scored positive window. Applying the 21-of-21 rule gives TN = 384, FP = 0, FN = 40, and TP = 344, with accuracy = 0.9479, recall = 0.8958, F1 = 0.9451, and MCC = 0.9007; both anomaly episodes are detected. In this 1.07 h nominal interval, eliminating persistent false positives requires about 200 s of confirmation, while 40 early anomalous windows are counted as negative during confirmation. Table 3 compares the raw classifier output with the primary and reviewer-proposed persistence settings.

### 3.4. Feature Relevance and Sensor-Dependence Risk

**Figure 5:**
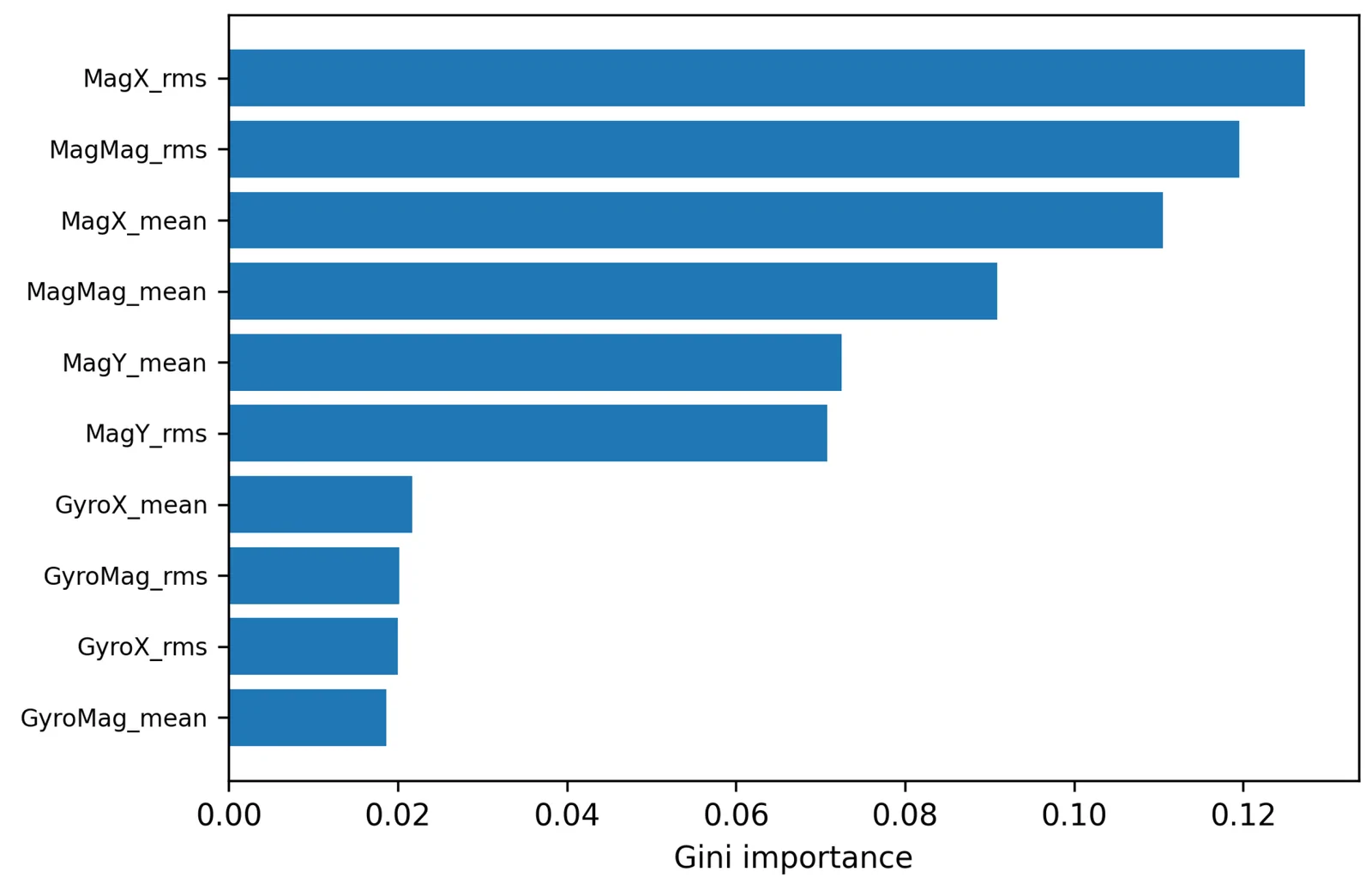
Gini importance from the exploratory 6000-window Random-Forest benchmark.

Magnetometer descriptors were initially considered. In the archived 6000-window exploratory RF analysis, the six largest Gini-importance values were magnetic descriptors (MagX_rms 0.1273, MagMag_rms 0.1195, MagX_mean 0.1105, MagMag_mean 0.0909, MagY_mean 0.0725, and MagY_rms 0.0708). Under validation-selected thresholds and the common 5-of-5 persistence layer, the gyroscope-only configuration achieves validation MCC = 0.9613 and final-test MCC = 0.9741. Acceleration only yields validation/test MCC = 0.4877/0.5526; acceleration+gyroscope 0.7511/0.9007; magnetometer only 0.2131/0.3460; and all nine channels 0.7984/0.9080. Thus, adding magnetic channels does not improve later-event performance in this split, and magnetometer-only is substantially weaker. The primary classifier is therefore gyroscope-only for empirical validation and to reduce installation dependence. The importance ranking is interpreted as predictive rather than causal. Figure 5 summarizes the exploratory feature-importance profile.

The comparison with Refs. [1,2] remains contextual because the published CASPER models use different windowing and validation rules. Under the fully specified comparator configurations, XGBoost performs similarly to the primary Random Forest after 5-of-5 persistence (MCC = 0.9767 versus 0.9741), whereas the fixed 1D-CNN and nominal autoencoder do not generate persistent final-test events. Because we evaluate only one fixed configuration per comparator family, these data are treated as controlled protocol-sensitivity evidence rather than a universal algorithm ranking.

### 3.5. PCA Geometry and Exploratory Disturbance Recordings

**Figure 6:**
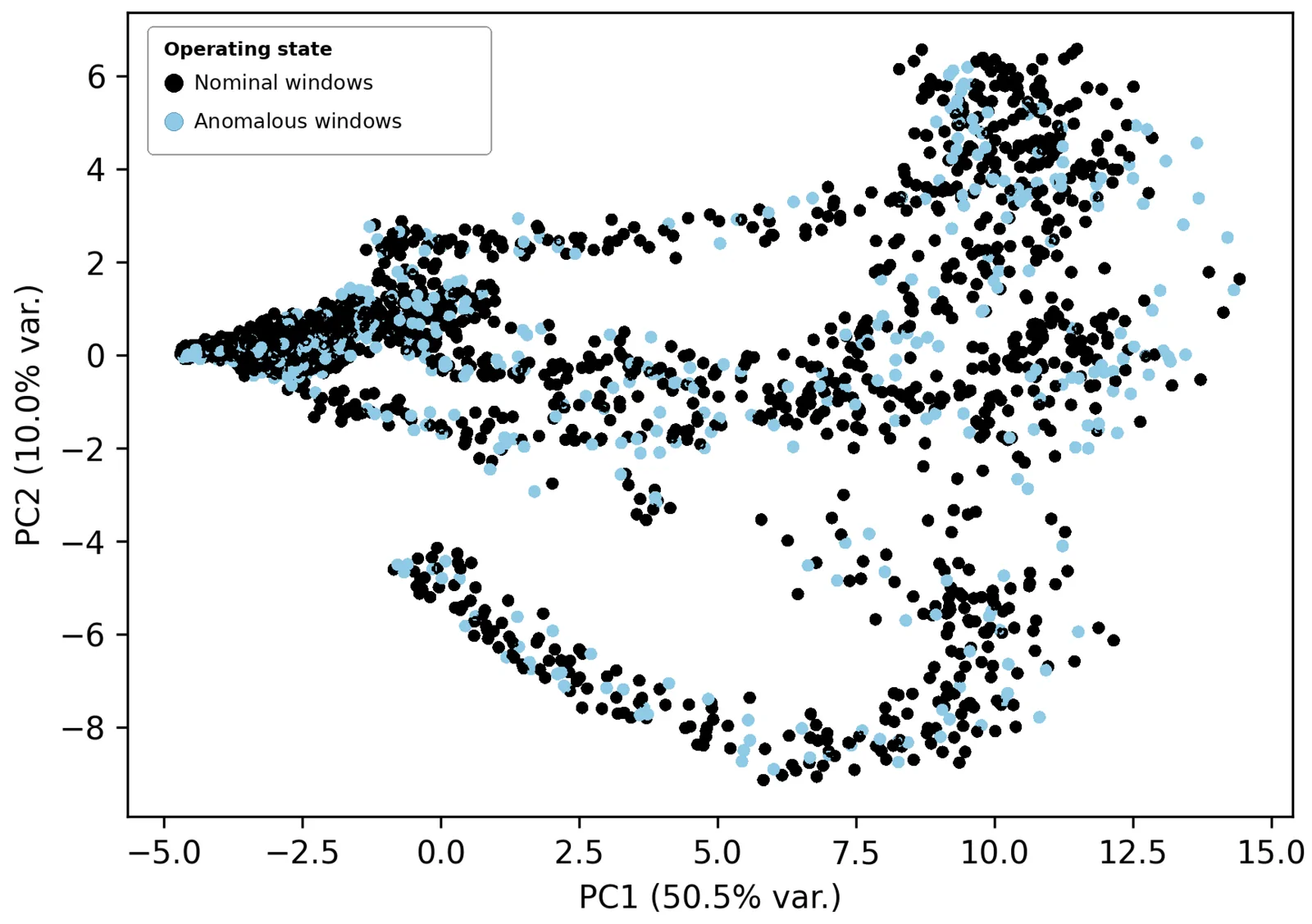
PCA projection of the exploratory 6000-window inertial feature set; nominal windows (black) and anomalous windows (light blue).

In the archived exploratory 6000-window analysis, PC1 and PC2 together retained 60.56% of feature variance (PC1 50.52% and PC2 10.04%). K-means applied in this two-dimensional representation yielded a silhouette coefficient of 0.753, whereas the adjusted Rand index relative to the binary labels was −0.002. Figure 6 therefore illustrates structured operating-regime geometry that does not match the supervised anomaly taxonomy. The five dedicated challenge recordings are discussed as heterogeneous disturbance examples, but the available results do not define or validate a PCA distance threshold, an OOD alarm rule, or an event-level persistence setting for them. They are not counted as five successful OOD detections.

The exploratory PCA geometry supports keeping visualization separate from supervised fault classification. The near-zero adjusted Rand index in Figure 6 shows that visually coherent regimes should not be relabeled as anomaly classes merely because they are separable. Magnetic disturbance, impacts, added mass, and vibration can represent unfamiliar contexts, but the current analysis does not convert those shifts into calibrated OOD alarms. Persistent or novel behavior should be reviewed before assigning component-level diagnosis.

### 3.6. Robustness, Ablation, and Protocol Results

**Figure 7:**
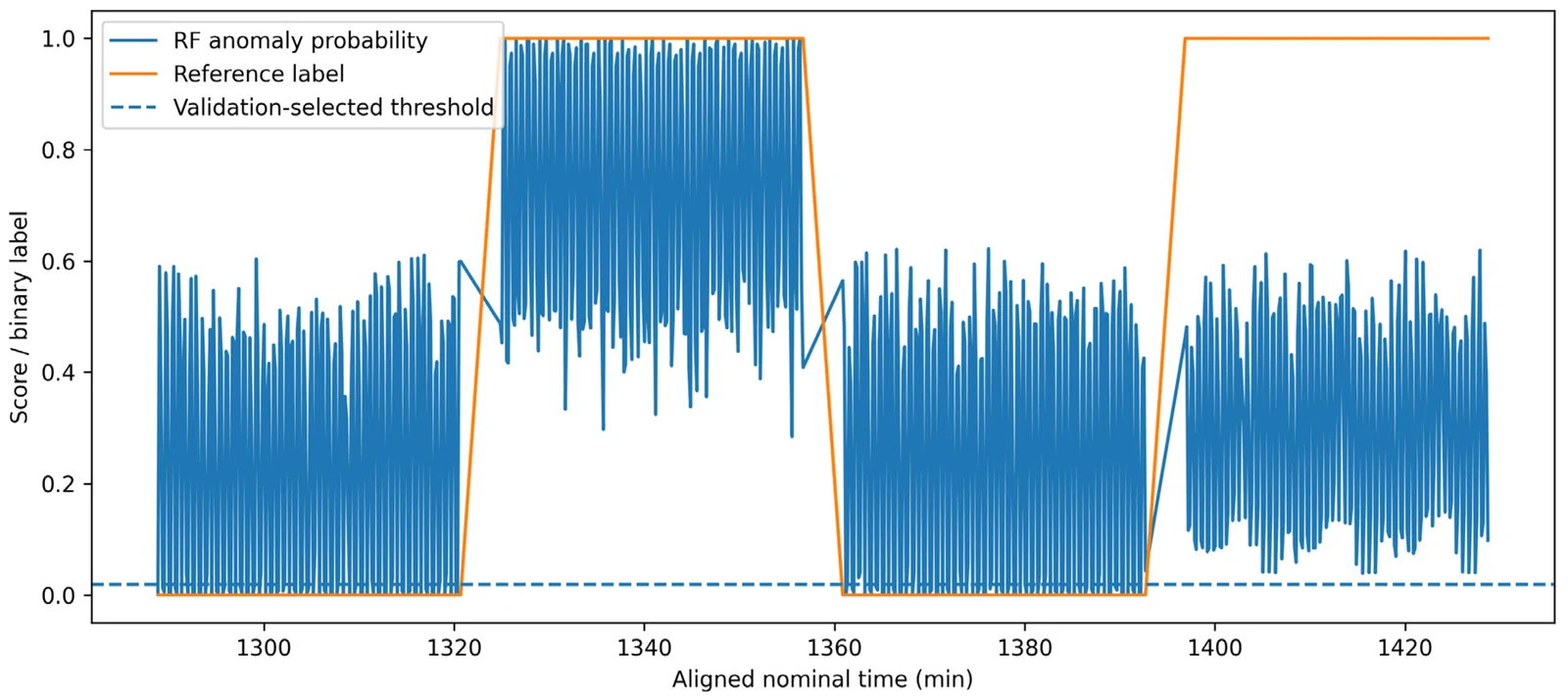
Final-test per-window RF probabilities, threshold, and reconstructed reference label.

The window RF probability sequence is shown in Figure 7. The validation-selected threshold is intentionally low because E5-E6 favor sensitivity; consequently, many nominal windows cross the raw threshold. Temporal persistence removes most isolated or short-lived crossings. This direct score trace demonstrates why the post-persistence confusion matrix must not be interpreted as classifier-only performance.

#### 3.6.1. Persistence Sensitivity

**Figure 8:**
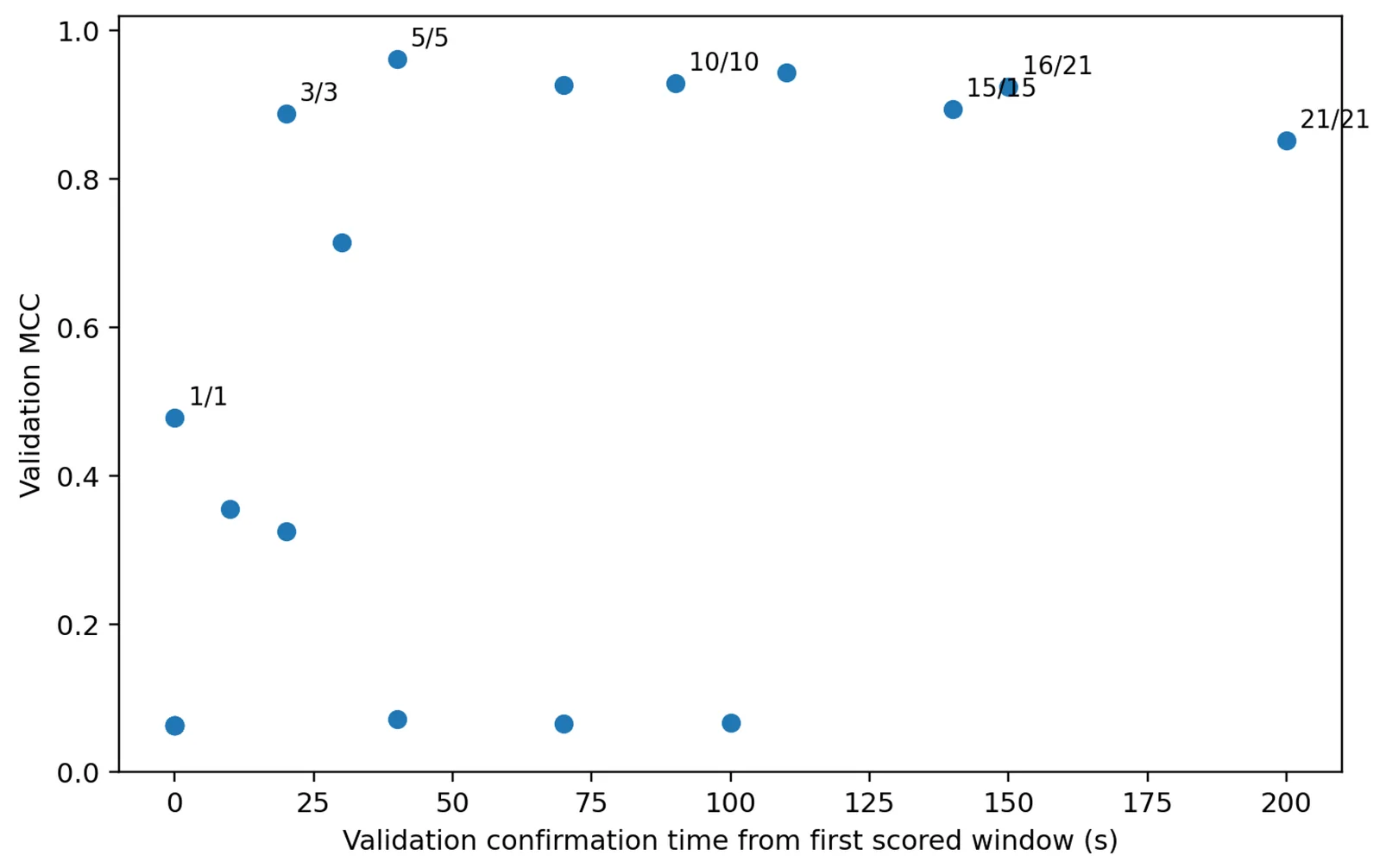
Validation MCC versus confirmation time for the H/K sweep.

The validation H/K sweep identifies 5-of-5 as the maximum-MCC operating point (validation MCC = 0.9613). More conservative all-of-K rules progressively reduce persistent false alarms but increase confirmation delay and early false negatives. On the final test, 10-of-10 yields zero persistent false-alarm events and MCC = 0.9542 with 90 s confirmation; 15-of-15 yields MCC = 0.9296 with 140 s confirmation; the 11-of-11 point has validation MCC = 0.9223 and final-test MCC = 0.9492, with zero false positives, 20 false negatives, and 100 s confirmation, and 21-of-21 yields MCC = 0.9007 with 200 s confirmation. Table 4 reports representative operating points. Figure 8 visualizes the validation trade-off between MCC and confirmation time.

#### 3.6.2. Guard-Band Sensitivity

**Figure 9:**
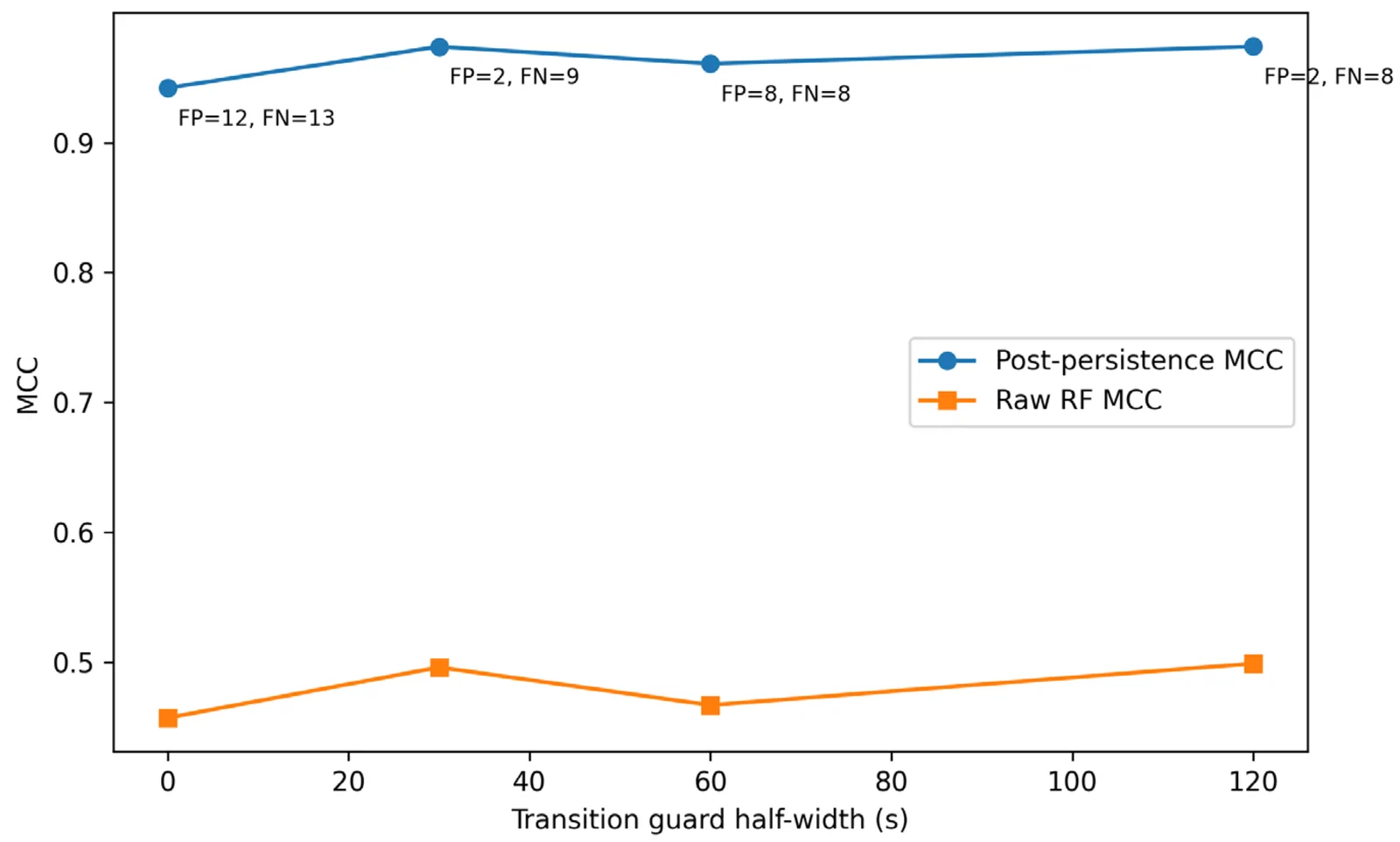
Final-test guard-band sensitivity of raw RF and post-persistence MCC.

Changing the guard width modifies both the development windows and the final scored set. Nevertheless, both final anomaly episodes remain detected for all tested guard widths. Post-persistence test MCC remains between 0.9421 and 0.9741. The 0 s setting yields 12 FP and 13 FN across 864 windows; the 30 s setting yields 2 FP and 9 FN; the 60 s setting yields 8 FP and 8 FN; and the primary 120 s setting yields 2 FP and 8 FN. The non-monotonic result argues against interpreting the 120 s exclusion as the sole source of high final performance. Table 5 reports the numerical guard-band results, and Figure 9 visualizes the corresponding MCC sensitivity.

#### 3.6.3. Sensor-Channel Ablation

**Figure 10:**
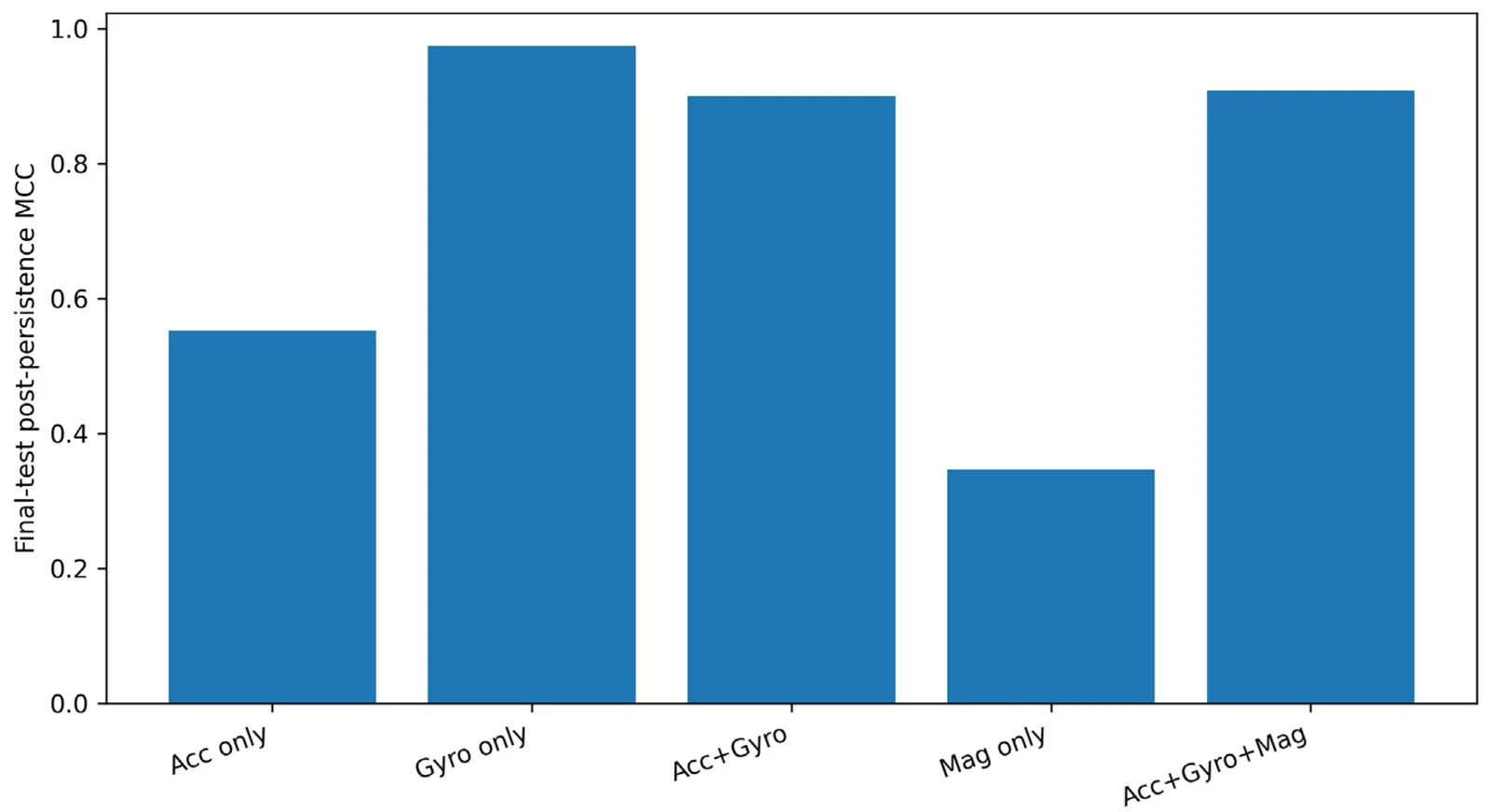
Final-test post-persistence MCC for the same-split sensor ablation.

The same-split ablation selects gyroscope-only on validation, not on the final test. It achieves validation/test post-persistence MCC of 0.9613/0.9741. All nine channels achieve 0.7984/0.9080, acceleration+gyroscope 0.7511/0.9007, acceleration only 0.4877/0.5526, and magnetometer only 0.2131/0.3460. The test ordering is broadly consistent with validation and supports the final gyroscope-only choice. The result also shows that the magnetic importance observed in the exploratory random-window analysis does not translate into the strongest later-event performance. Table 6 reports the channel-specific results, while Figure 10 visualizes final-test post-persistence MCC.

#### 3.6.4. Rolling-Origin Event Evaluation

The rolling-origin analysis demonstrates that the strong E7-E8 result is not uniformly reproduced on every earlier held-out event. With the gyroscope-only pipeline and validation-specific thresholds, Fold A (test E4) reaches post-persistence MCC = 0.9296 and detects the event with 40 s confirmation. Fold B (test E5) produces an all-negative persistent decision (MCC = 0) and misses the event. Fold C (test E6) reaches MCC = 0.2465 and detects the event only after 130 s. These results materially strengthen the limitation statement: two successful final events cannot support a claim of stable generalization across all velocity-deviation episodes. Table 7 summarizes the three rolling-origin folds and their event-level outcomes.

#### 3.6.5. Same-Split Model Check

**Figure 11:**
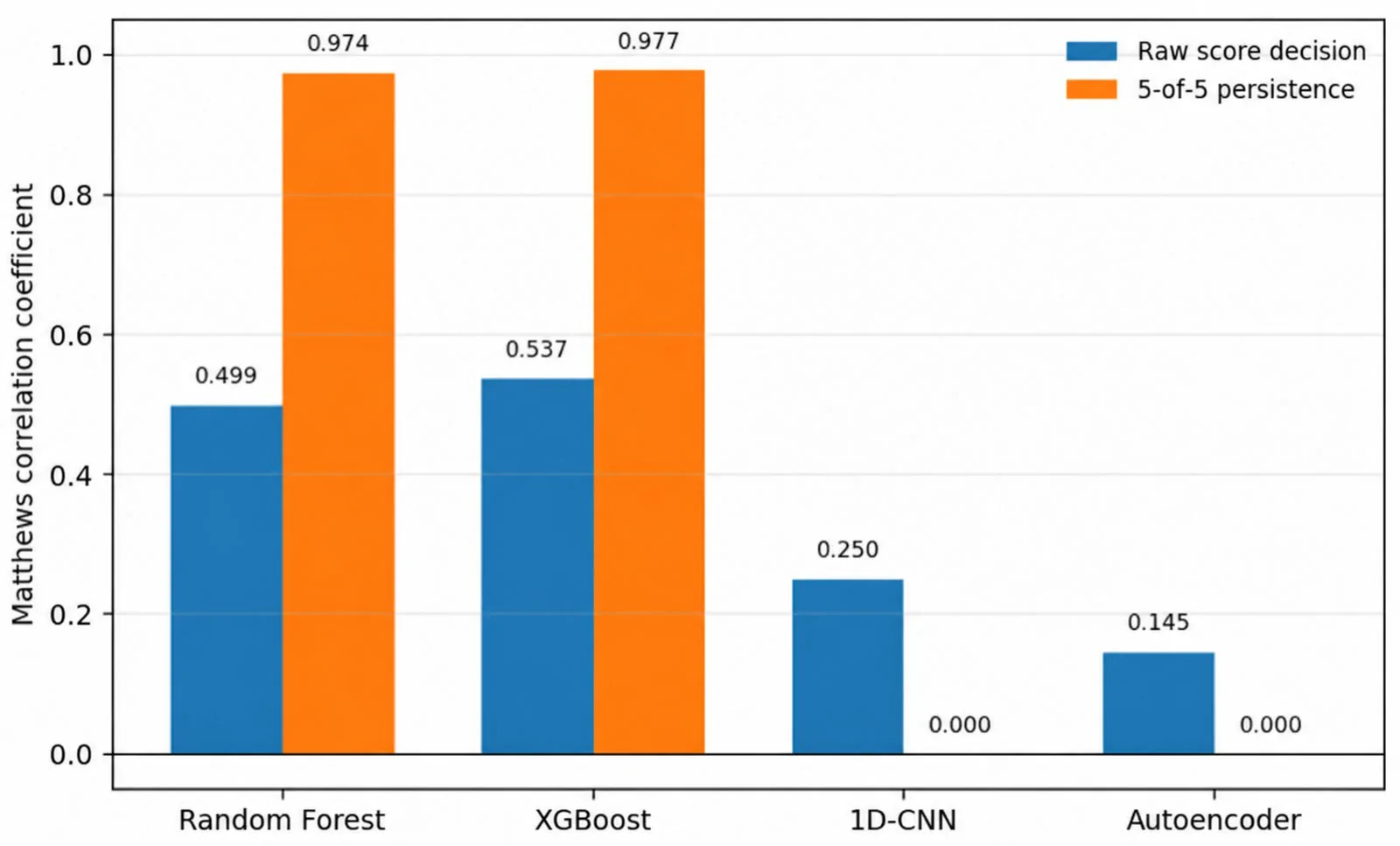
Final-test MCC for fully specified same-split comparators before and after persistence.

In the E1–E4/E5–E6/E7–E8 split, the primary Random Forest reaches raw/test MCC = 0.4988 and 5-of-5 MCC = 0.9741. The fully specified XGBoost reaches raw MCC = 0.5372 and 5-of-5 MCC = 0.9767, with one persistent false-positive window, eight false-negative windows, and both test events detected. The fixed 1D-CNN obtains test ROC-AUC = 0.6147 and PR-AUC = 0.6978 with raw MCC = 0.2498, but no positive sequence survives the common 5-of-5 rule. The nominal-trained autoencoder obtains ROC-AUC = 0.6914 and PR-AUC = 0.7005 with raw MCC = 0.1451 and likewise produces no persistent test event. Table 8 and Figure 11 summarize the raw and post-persistence comparator results. These fully documented runs show that XGBoost is comparable to RF under the same chronology, whereas the fixed neural and reconstruction baselines are less effective under the common decision rule. The comparison is not interpreted as a universal algorithm ranking because only one robot/domain and one fixed comparator configuration per model family are evaluated.

#### 3.6.6. Protocol-Component Ablation

Table 9 formalizes the protocol ablation. Removing persistence produces the largest degradation (MCC 0.9741→0.4988), confirming that temporal decision logic is a major component of the deployed detector. Replacing 5-of-5 with 21-of-21 lowers MCC to 0.9007 and increases confirmation from 40 s to 200 s. Removing the ±120 s guard reduces MCC to 0.9421 but preserves detection of both final events, so transition exclusion alone does not create the result. Replacing gyroscope-only inputs with all nine axes or acceleration+gyroscope reduces MCC to 0.9080 and 0.9007, respectively. The five-seed random-window audit is shown separately because it changes the estimand and is not a paired final-test ablation. Removing persistence reduces MCC by 0.4753 (0.9741 to 0.4988), while the more conservative 21-of-21 rule yields MCC = 0.9007 and increases confirmation delay from 40 s to 200 s. The matched model-family check shows that XGBoost is comparable to Random Forest after persistence (MCC = 0.9767 versus 0.9741); therefore, the measurable benefit is attributed to the validated decision protocol and channel configuration.

The random-window row reports the five-seed raw-classifier mean and is not a paired event-level ablation. Its 26.6 mean false-positive and 98.8 mean false-negative windows are averaged across 1239-window test sets; persistence and confirmation time are not defined for these scattered rows. The same RF, feature, scaling, and validation-threshold procedure is used, while the split alone changes. The results describe the partitioning estimand and are not evidence of generalization to new events.

### 3.7. Safeguards and Validity Scope

Table 10 summarizes the safeguards used in the final analysis. They reduce distinct sources of interpretation: event leakage, transition uncertainty, future-data fitting, installation-sensitive channels, and isolated threshold crossings. Channel selection and persistence are supported by same-split validation analyses.

## 4. Discussion

The principal finding is methodological. The RF vector shows that the final result comes from the interaction between a sensitive classifier and an explicitly tuned temporal decision layer. The validation-selected 5-of-5 rule raises specificity to 0.9948 and MCC to 0.9741 while retaining recall = 0.9792. The legacy 21-of-21 rule eliminates the two post-persistence false-positive windows and exactly reproduces the originally reported zero-FP confusion matrix, but reduces recall to 0.8958 and increases confirmation delay from approximately 40 s to 200 s. The observed 5-of-5 false-alarm rate of 0.9375 events/h is based on 1.0667 h of nominal held-out exposure and does not provide a reliable long-term estimate. The positioning is consistent with recent robot-monitoring research. Supervised and hybrid approaches can provide high discrimination when fault examples are represented [4,16,17,18,19,20,21], while autoencoder, transformer, graph, and variational methods improve representation capacity for complex multivariate data [10,14,22,23,24,31,32,33,34]. The present framework occupies a different point in the design space: it prioritizes traceability, low inference complexity, explicit context handling, and a validation protocol that is difficult to game through correlated windows. Related human–robot collaboration studies show that multimodal and sequence-aware anomaly models can add representational capacity when richer interaction data are available [35,36]. In parallel, sensor-data predictive maintenance, remaining-useful-life research, and robot-gearbox reliability studies emphasize that condition classification, prognosis, and use-condition uncertainty are distinct validation problems [37,38,39]. Domain-generalization and multi-source adaptation studies in rotating machinery further show why operating-condition shift deserves explicit treatment rather than being absorbed into a single fixed decision boundary [40,41,42]. 

### 4.1. Synchronization Uncertainty and Guard-Band Evaluation

Alignment uncertainty is central because the public CASPER materials document different effective sampling behavior and an explicit label-reconstruction shift [1,2]. The ±120 s exclusion zone remains the primary synchronization safeguard, but the new sensitivity analysis shows that the conclusion is not created solely by removing two minutes around each transition. With validation-specific thresholds and 5-of-5 persistence, the final-test MCC values are 0.9421, 0.9739, 0.9608, and 0.9741 for guard half-widths of 0, 30, 60, and 120 s, respectively; both held-out anomaly episodes are detected in every setting. The corresponding FP/FN counts are 12/13, 2/9, 8/8, and 2/8. The relationship is not monotonic, indicating that guard width changes both the development distribution and the scored transition content. Even the 0 s analysis does not establish exact physical onset latency because the public sensor/controller alignment remains approximate. The primary claim is therefore robust steady-state later-event recognition, with explicit evidence that performance persists when less transition data are removed.

### 4.2. Sensor Selection and Physical Interpretability

The sensor ablation resolves the earlier ambiguity between predictive importance, causal interpretation, and portability. Gyroscope-only is selected on validation (post-persistence MCC = 0.9613) and obtains the highest final-test MCC = 0.9741. Adding acceleration reduces validation/test MCC to 0.7511/0.9007, while adding magnetic channels to acceleration+gyroscope yields 0.7984/0.9080. Magnetometer-only performs substantially worse (0.2131/0.3460). Therefore, the primary sensor decision is no longer justified only by anticipated portability: it is supported by an identical-split quantitative ablation. At the same time, the exploratory magnetic Gini importance remains scientifically useful because it shows how a locally discriminative channel can encode environmental context without improving later-event generalization. The present result should not be generalized to other mounts or workcells without independent validation. The sensor-related result identifies a potential installation-sensitive shortcut and defines a configuration for future cross-platform replication. Figure 6 provides a complementary unsupervised visualization: the PCA feature space exhibits coherent geometry, but its near-zero adjusted Rand index confirms that these regimes do not reproduce the binary anomaly labels. The PCA plot is descriptive and does not alter the RF decision. Any future context correction or OOD alarm should be reported only after its variables, threshold, calibration data, and alarm logic are specified and evaluated on data excluded from model development.

### 4.3. Same-Protocol Model Check and Relation to Published CASPER Outcomes

Published results in [1,2] remain literature context rather than a direct ranking because their split, window, transition, and decision rules differ from the present protocol. The benchmark uses fully specified and reproducible comparator configurations. XGBoost is close to the primary RF after the common 5-of-5 rule (MCC 0.9767 versus 0.9741), whereas the fixed 1D-CNN and nominal autoencoder retain moderate raw ranking ability but generate no persistent test event. The result does not establish tree-model superiority; rather, it shows that event-held-out performance depends jointly on score calibration, representation, and temporal decision logic. Because the benchmark evaluates only one fixed configuration per comparator family, it is deliberately interpreted as a controlled sensitivity analysis rather than an exhaustive architecture competition. A separate CatBoost-based study has also evaluated the public CASPER dataset [43].

### 4.4. Limitations and Threats to Validity

The principal limitation is the small number of independent anomaly episodes: E7-E8 are two complete episodes from one UR3e and one acquisition domain, and both are detected in the final test. This 2/2 result is a feasibility observation, not a reliable estimate of event-level sensitivity or cross-condition generalization. The rolling-origin analysis reinforces this limit: the 5-of-5 detector detects E4 (MCC = 0.9296; 40 s), misses E5 (MCC = 0), and detects E6 weakly (MCC = 0.2465; 130 s). Nominal exposure is 1.0667 h; one persistent false-alarm event under 5-of-5 corresponds to 0.9375 events/h, and zero observed events under 21-of-21 does not establish long-term reliability. The five challenge recordings remain qualitative disturbance examples, not a calibrated OOD benchmark. Future experimental campaigns will extend nominal exposure across multiple days and collect additional independent anomaly episodes across payload, mounting, and robot conditions.

## 5. Conclusions

This study presents a leakage-aware external-IMU anomaly-monitoring procedure evaluated on the public CASPER collaborative-robot record. The contribution is an experimentally validated architecture, not a new learning algorithm. The evaluation includes quantitative per-window RF scores, same-split sensor ablation, quantified H/K and guard-band sensitivity, rolling-origin event checks, a formal one-factor protocol ablation, and fully specified alternative model families under the same event partition. Validation selects a 31-feature gyroscope-only RF representation, threshold 0.018884, and 5-of-5 persistence. On E7–E8, raw RF yields TN = 153, FP = 231, FN = 0, TP = 384 (MCC = 0.4988), whereas 5-of-5 yields TN = 382, FP = 2, FN = 8, TP = 376 (MCC = 0.9741). The 11-of-11 setting gives TN = 384, FP = 0, FN = 20, TP = 364 (recall = 0.9479; MCC = 0.9492) with 100 s confirmation; the 21-of-21 setting reproduces the zero-FP matrix with 200 s confirmation and recall = 0.8958. Random-window results use the same classifier and validation-selection procedure but mix events across partitions, and therefore do not establish later-event generalization. The state-reset policy is annotation-aware and retrospective, so continuous label-independent online alarming remains to be evaluated. The evidence supports event recognition within the same robot/acquisition domain. The scientific novelty is methodological: the study quantifies how event isolation, boundary uncertainty, sensor choice, and temporal persistence change the interpretation of a robot record.

## Figures and Tables

**Table 1:** Data sources used in the revised leakage-aware analysis.

Dataset Element	Value	Role in This Study
Primary robot	Universal Robots UR3e	Source robot/single acquisition domain
External sensor	Nicla Sense ME	9-axis IMU acquisition
Raw IMU rows	1,751,682 valid rows	Public external-sensor record
Aligned analysis record	1,728,006 samples	Published alignment/truncation basis
Primary supervised channels	Gyroscope only (31 features)	Selected on validation by same-split ablation
Alternative channel sets	Acc; Acc+Gyro; Mag; Acc+Gyro+Mag	Same-split sensor ablation
External challenge files	5 disturbance families	Exploratory PCA visualization

**Table 2:** Chronological partition and fixed primary evaluation settings.

Protocol Value	Training	Validation	Held-Out Test	Leakage Control	Purpose
Velocity events	E1–E4	E5–E6	E7–E8	No event split across sets	Later-event evaluation
Window	200 samples	200 samples	200 samples	Non-overlapping	~10 s descriptive unit
Primary guard	±120 s	±120 s	±120 s	Guard windows omitted; not negative votes; state resets at retained-segment gaps	Alignment uncertainty
Primary sensor set	Gyroscope only	Gyroscope only	Gyroscope only	Selected without test	31-feature motion evidence
Scaling	Fit	Frozen	Frozen	Training only	Prevent future-data fitting
RF	100 trees; depth 8	Threshold selected	Frozen	Test unseen	Supervised score
Threshold	—	0.018884 selected by MCC	0.018884 frozen	Validation only	Raw-window decision
Persistence	Candidate H/K	5-of-5 selected	5-of-5 frozen	Validation only	Operational confirmation
Eligible windows	6676 windows	768 windows	768 core windows (384 nominal + 384 anomalous); 43 post-E8 rows outside core	Event hold-out; per-window manifest available on request	Core-test identities and tail exclusion specified

**Table 3:** Raw RF and post-persistence final-test performance.

Configuration	TN/FP/FN/TP	Accuracy	Precision	Recall	F1	MCC	Confirmation
Initial RF	153/231/0/384	0.6992	0.6244	1.0000	0.7688	0.4988	0 s
5-of-5	382/2/8/376	0.9870	0.9947	0.9792	0.9869	0.9741	40 s
11-of-11	384/0/20/364	0.9740	1.0000	0.9479	0.9733	0.9492	100 s
21-of-21	384/0/40/344	0.9479	1.0000	0.8958	0.9451	0.9007	200 s

**Table 4:** Representative H/K operating points; selection of the primary setting uses validation data.

H/K	Val. MCC	Test MCC	Test FP	Test FN	FA Events/h	Confirmation
1/1	0.4778	0.4988	231	0	94.6875	0 s
3/3	0.8881	0.9160	29	4	24.3750	20 s
5/5	0.9613	0.9741	2	8	0.9375	40 s
10/10	0.9296	0.9542	0	18	0.0000	90 s
11/11	0.9223	0.9492	0	20	0.0000	100 s
15/15	0.8936	0.9296	0	28	0.0000	140 s
21/21	0.8520	0.9007	0	40	0.0000	200 s

**Table 5:** Final-test guard-band sensitivity with validation-specific thresholds.

Guard	N	Accuracy	MCC	FP	FN	FA Events/h
0 s	864	0.9711	0.9421	12	13	3.3333
30 s	840	0.9869	0.9739	2	9	0.8571
60 s	816	0.9804	0.9608	8	8	3.5294
120 s	768	0.9870	0.9741	2	8	0.9375

**Table 6:** Same-split sensor-channel ablation with channel-specific validation thresholds.

Sensor Set	Features	Val. MCC	Test MCC	Test Accuracy	Test FP/FN	ROC-AUC
Acceleration	31	0.4877	0.5526	0.7747	66/107	0.6001
Gyroscope	31	0.9613	0.9741	0.9870	2/8	0.7413
Acc+Gyro	62	0.7511	0.9007	0.9479	0/40	0.7177
Mag only	31	0.2131	0.3460	0.6445	242/31	0.6312
All 9 axes	93	0.7984	0.9080	0.9531	6/30	0.7321

**Table 7:** Rolling-origin chronological evaluation of the gyroscope-only RF.

Fold	Train	Validation	Test	Threshold	Post-Persist MCC	Event/ Confirmation
A	E1–E2	E3	E4	0.275	0.9296	Detected/40 s
B	E1–E3	E4	E5	0.400	0.0000	Missed
C	E1–E4	E5	E6	0.075	0.2465	Detected/130 s

**Table 8:** Same-split model comparison on the final chronological test.

Model	Threshold	Raw MCC	5/5 MCC	ROC-AUC	PR-AUC	Persistent Events
Random Forest	0.018884	0.4988	0.9741	0.7413	0.7520	2/2
XGBoost	0.007925	0.5372	0.9767	0.7299	0.7460	2/2
1D-CNN	0.509585	0.2498	0.0000	0.6147	0.6978	0/2
Autoencoder	0.074613	0.1451	0.0000	0.6914	0.7005	0/2

**Table 9:** One-factor protocol ablation relative to the primary event-held-out configuration.

Configuration	Changed Component	Accuracy	MCC	FP/FN	Confirmation
Primary protocol	Reference	0.9870	0.9741	2/8	40 s
No persistence	Remove temporal filtering	0.6992	0.4988	231/0	0 s
Legacy 21-of-21	Change persistence	0.9479	0.9007	0/40	200 s
No transition guard	Guard: ±120 s → 0 s	0.9711	0.9421	12/13	40 s
All nine axes	Gyro → Acc+Gyro+Mag	0.9531	0.9080	6/30	—
Acceleration+gyroscope	Gyro → Acc+Gyro	0.9479	0.9007	0/40	—
Random-window audit (raw RF; 5 seeds)	70/15/15 stratified split; otherwise, same RF pipeline	0.8988	0.6392	26.6/98.8	—

**Table 10:** Leakage-control and reproducibility safeguards in the final analysis.

Safeguard	Implementation	Why it Matters
Chronological event hold-out	E7-E8 never used in development	Tests later events; prevents window leakage
Transition uncertainty	Primary ±120 s guard; 0/30/60/120 s sensitivity	Quantifies alignment/guard dependence
Non-overlapping windows	200-sample windows; 200-sample stride	Avoids overlap; dependence remains
Training-only transformations	Scaler + RF fitted before final test	Prevents future-data leakage
Sensor-channel selection	Five same-split channel sets; gyro selected on validation	Quantifies shortcut/portability risk
Probability threshold	0.018884 on E5–E6 (MCC)	Prevents test-set threshold tuning
Temporal persistence	20 H/K candidates; validation 5/5	Exposes false-alarm/latency trade-off
Legacy consistency check	21/21 reproduces original matrix	Separates classifier from conservative decision layer
Challenge recordings	Five heterogeneous disturbance files described qualitatively; no calibrated PCA threshold or OOD detection count is claimed	Avoids assigning one fault label to distinct disturbances

## Data Availability

The raw CASPER data analyzed in this study are publicly available in the Kaggle dataset ‘Industrial Robotic Arm—IMU Data (CASPER 1 & 2)’ and in the associated CASPER resources cited in References [1,2]. The corresponding author will make the analysis-generated per-window manifest, including raw RF probabilities, labels, timestamps, and retained-segment identifiers, available upon request.

## References

[1] Kayan H., Heartfield R., Rana O., Burnap P., Perera C. (2024). CASPER: Context-Aware IoT Anomaly Detection System for Industrial Robotic Arms. ACM Trans. Internet Things.

[2] Kayan H., Rana O., Burnap P., Perera C. CASPER: Context-Aware Anomaly Detection System for Industrial Robotic Arms. Proceedings of the 2023 IEEE International Conference on Pervasive Computing and Communications Workshops and Other Affiliated Events (PerCom Workshops).

[3] Iqbal M., Suhail S., Freudenthaler S., Fahim M., Matulevicius R. (2026). TWINE: ISO 23247-compliant digital twin and explainable AI framework for anomaly detection. Internet Things.

[4] Polenghi A., Cattaneo L., Macchi M. (2024). A framework for fault detection and diagnostics of articulated collaborative robots based on hybrid series modelling of Artificial Intelligence algorithms. J. Intell. Manuf..

[5] Kermenov R., Nabissi G., Longhi S., Bonci A. (2023). Anomaly Detection and Concept Drift Adaptation for Dynamic Systems: A General Method with Practical Implementation Using an Industrial Collaborative Robot. Sensors.

[6] Ayankoso S., Gu F., Louadah H., Fahham H., Ball A. (2024). Artificial-Intelligence-Based Condition Monitoring of Industrial Collaborative Robots: Detecting Anomalies and Adapting to Trajectory Changes. Machines.

[7] Sabry A.H., Ungku Amirulddin U.A.B. (2024). A review on fault detection and diagnosis of industrial robots and multi-axis machines. Results Eng..

[8] Kumar P., Khalid S., Kim H.S. (2023). Prognostics and Health Management of Rotating Machinery of Industrial Robot with Deep Learning Applications—A Review. Mathematics.

[9] Mallioris P., Aivazidou E., Bechtsis D. (2024). Predictive maintenance in Industry 4.0: A systematic multi-sector mapping. CIRP J. Manuf. Sci. Technol..

[10] Yun H., Kim H., Jeong Y.H., Jun M.B.G. (2023). Autoencoder-based anomaly detection of industrial robot arm using stethoscope based internal sound sensor. J. Intell. Manuf..

[11] Zhong Z., Zhao Y., Yang A., Zhang H., Qiao D., Zhang Z. (2022). Industrial Robot Vibration Anomaly Detection Based on Sliding Window One-Dimensional Convolution Autoencoder. Shock Vib..

[12] Park Y.S., Yoo D.Y., Lee J.W. (2021). Programmable Motion-Fault Detection for a Collaborative Robot. IEEE Access.

[13] Kumar P., Raouf I., Kim H.S. (2024). Transfer learning for servomotor bearing fault detection in the industrial robot. Adv. Eng. Softw..

[14] Yang B., Long W., Zhang Y., Xi Z., Jiao J., Li Y. (2024). Multivariate time series anomaly detection: Missing data handling and feature collaborative analysis in robot joint data. J. Manuf. Syst..

[15] Wang S., Tao J., Jiang Q., Chen W., Qin C., Liu C. (2024). A digital twin framework for anomaly detection in industrial robot system based on multiple physics-informed hybrid convolutional autoencoder. J. Manuf. Syst..

[16] Fedorova D., Tlach V., Kuric I., Dodok T., Zajačko I., Tucki K. (2025). Technical Diagnostics of Industrial Robots Using Vibration Signals: Case Study on Detecting Base Unfastening. Appl. Sci..

[17] Lu K., Chen C., Wang T., Cheng L., Qin J. (2022). Fault diagnosis of industrial robot based on dual-module attention convolutional neural network. Auton. Intell. Syst..

[18] Pan J., Qu L., Peng K. (2022). Deep residual neural-network-based robot joint fault diagnosis method. Sci. Rep..

[19] Liu Q., Yu Y., Han B.S., Zhou W. (2025). An evolutionary deep learning approach using flexible variable-length dynamic stochastic search for anomaly detection of robot joints. Appl. Soft Comput..

[20] Niu Q., Sui Z., Han J., Zhao Y. (2025). An Industrial Robot Gearbox Fault Diagnosis Approach Using Multi-Scale Empirical Mode Decomposition and a One-Dimensional Convolutional Neural Network-Bidirectional Gated Recurrent Unit Method. Processes.

[21] Wu J., Zhang Y., Gao B., Xia L., Zhu X., Wang H., Wan X. (2025). A Hybrid Deep Learning Framework for Enhanced Fault Diagnosis in Industrial Robots. Algorithms.

[22] Choi K., Yi J., Park C., Yoon S. (2021). Deep Learning for Anomaly Detection in Time-Series Data: Review, Analysis, and Guidelines. IEEE Access.

[23] Pang G., Shen C., Cao L., van den Hengel A. (2021). Deep Learning for Anomaly Detection: A Review. ACM Comput. Surv..

[24] Ruff L., Kauffmann J.R., Vandermeulen R.A., Montavon G., Samek W., Kloft M., Dietterich T.G., Müller K.R. (2021). A Unifying Review of Deep and Shallow Anomaly Detection. Proc. IEEE.

[25] Garg A., Zhang W., Samaran J., Savitha R., Foo C.S. (2022). An Evaluation of Anomaly Detection and Diagnosis in Multivariate Time Series. IEEE Trans. Neural Netw. Learn. Syst..

[26] Kim S., Choi K., Choi H.S., Lee B., Yoon S. (2022). Towards a Rigorous Evaluation of Time-Series Anomaly Detection. Proc. AAAI Conf. Artif. Intell..

[27] Paparrizos J., Boniol P., Palpanas T., Tsay R.S., Elmore A.J., Franklin M.J. (2022). Volume Under the Surface: A New Accuracy Evaluation Measure for Time-Series Anomaly Detection. Proc. VLDB Endow..

[28] Saito T., Rehmsmeier M. (2015). The precision-recall plot is more informative than the ROC plot when evaluating binary classifiers on imbalanced datasets. PLoS ONE.

[29] Breiman L. (2001). Random Forests. Mach. Learn..

[30] Jolliffe I.T., Cadima J. (2016). Principal component analysis: A review and recent developments. Philos. Trans. R. Soc. A.

[31] Azzalini D., Bonali L., Amigoni F. (2021). A Minimally Supervised Approach Based on Variational Autoencoders for Anomaly Detection in Autonomous Robots. IEEE Robot. Autom. Lett..

[32] Tuli S., Casale G., Jennings N.R. (2022). TranAD: Deep Transformer Networks for Anomaly Detection in Multivariate Time Series Data. Proc. VLDB Endow..

[33] Deng A., Hooi B. (2021). Graph Neural Network-Based Anomaly Detection in Multivariate Time Series. Proc. AAAI Conf. Artif. Intell..

[34] Huang Y., Yang B., Wang S., Xie K., Wang Y., Yi L., Dong N. (2025). Attentive Neural Processes Based on Reliable Inferences for Industrial Equipment Anomaly Detection. Comput. Ind..

[35] Yang L., Yan W., Wu H. (2021). Comparison of deep learning-based methods in multimodal anomaly detection: A case study in human–robot collaboration. Sci. Prog..

[36] Schirmer F., Kranz P., Schmitt J., Kaupp T. (2023). Anomaly detection for dynamic human-robot assembly: Application of an LSTM-based autoencoder to interpret uncertain human behavior in HRC. Companion of the 2023 ACM/IEEE International Conference on Human-Robot Interaction.

[37] Fordal J.M., Schjølberg P., Helgetun H., Skjermo T.Ø., Wang Y., Wang C. (2023). Application of sensor data based predictive maintenance and artificial neural networks to enable Industry 4.0. Adv. Manuf..

[38] Taşcı B., Omar A., Ayvaz S. (2023). Remaining useful lifetime prediction for predictive maintenance in manufacturing. Comput. Ind. Eng..

[39] Park J.G., Yoon H., Youn B.D. (2023). Probabilistic framework for reliable optimal design of gearboxes in general-purpose industrial robots considering random use conditions. J. Comput. Des. Eng..

[40] Gao T., Yang J., Wang W., Fan X. (2024). A domain feature decoupling network for rotating machinery fault diagnosis under unseen operating conditions. Reliab. Eng. Syst. Saf..

[41] Gao T., Yang J., Tang Q. (2024). A multi-source domain information fusion network for rotating machinery fault diagnosis under variable operating conditions. Inf. Fusion.

[42] Li Y., Yang J., Wang W., Gao T. (2026). A joint collaborative adaptation network for fault diagnosis of rolling bearing under class imbalance and variable operating conditions. Adv. Eng. Inform..

[43] Doshi P., Daftary N., Jani H., Nair A. (2026). CatBoost-Driven Anomaly Detection in Industrial Robotic Arms Using CASPER Dataset. IEEE Sens. Lett..

